# The effect of ketamine on preventing postpartum depression

**DOI:** 10.25122/jml-2020-0116

**Published:** 2021

**Authors:** Mina Alipoor, Marzeyeh Loripoor, Majid Kazemi, Farshid Farahbakhsh, Ali Sarkoohi

**Affiliations:** 1. Department of Anesthesiology, School of Paramedical Sciences, Rafsanjan University of Medical Sciences, Rafsanjan, Iran; 2. Department of Reproductive Health and Midwifery, School of Nursing and Midwifery; Geriatric Care Research Center, Rafsanjan University of Medical Sciences, Rafsanjan, Iran; 3. Department of Medical Surgical Nursing, Faculty of Nursing and Midwifery, Non-Communicable Disease Research Center, Rafsanjan University of Medical Sciences, Rafsanjan, Iran; 4. Department of Anesthesiology, School of Medicine, Rafsanjan University of Medical Sciences, Rafsanjan, Iran

**Keywords:** ketamine, postpartum depression, caesarian section, general anesthesia, prevention

## Abstract

Postpartum depression is a common disabling psychosocial disorder that could have adverse effects on the life of the mother, infant, and family. The present study was conducted to evaluate the effect of ketamine on preventing postpartum depression in women undergoing caesarian sections considering the relatively known positive effect of ketamine on major depression. The present double-blind, randomized clinical trial was conducted on 134 women undergoing scheduled caesarian sections. Participants were randomly allocated into two groups of control and intervention. To induce anesthesia, 1–2 mg/kg of body weight of Nesdonal and 0.5 mg/kg of body weight of ketamine were used in the intervention group, while only 3–5 mg/kg of body weight Nesdonal was administered in the control group. Data were gathered using the Edinburgh Postnatal Depression Scale (EPDS) in three stages: before the caesarian section and two and four weeks after the caesarian section. Data were analyzed using variance analysis with repeated measures and the Chi-square test. Results of the present study showed that the mean (± standard deviation) of the depression score in the intervention and control groups were 13.78±3.87 and 13.79±4.78(p = 0.98) before the caesarian section, 11.82±3.41 and 14.34±4.29 (p < 0.001) two weeks after and 10.84±3.48 and 13.09±3.79 (p = 0.001) four weeks after the caesarian section, respectively. Using ketamine in the induction of general anesthesia could be effective in preventing postpartum depression. However, further studies are required to strengthen these findings.

## Introduction

Depression is the most common complication of delivery with a prevalence of 10% to 15% and is more common than gestational diabetes (3–8%) and preterm delivery (12.3%) [1]. Major depression, which requires hospitalization, occurs more frequently after birth than in any other period of life in women [2]. The World Health Organization has estimated that this disorder would be the second cause of the global disease burden in 2020. In the conducted evaluations, the prevalence of postpartum depression has been reported between 25% and 42.1% in Iran [3].

Postpartum depression has destructive effects on the mother, neonate, and family [4]. It would expose the mother to the risk of social isolation due to the lack of energy, exhaustion and the feeling of disability, worthlessness, and disappointment [5]. Fifty percent of the mothers think about killing themselves or their neonates due to postpartum depression [6, 7]. On the other hand, 24% to 50% of the husbands of depressed mothers also suffer from depression [8]. Following postpartum depression, conflicts, quarrels, and discontinuing couple’s social support for each other, separation and divorce would threaten the couple’s married life [9–11].

Postpartum depression affects the mother-neonate relationship all around the world; despite various cultures and socioeconomic statuses, the suffering mothers are less sensitive and responding to their neonates. Neonatal care such as feeding the neonate - breastfeeding, routine sleeping, regular visits to the physician, vaccination, and providing the safety of the neonate is decreased in these mothers [12]. Also, studies have indicated the negative effects of postpartum depression on cognitive and emotional growth during the neonatal period and afterward [13, 14].

A significant number of patients do not seek treatment due to various reasons such as feeling ashamed for not being happy when they are expected to be happy, being labeled as a mental health patient, and unawareness; and when they seek treatment, they mostly prefer not to consume psychotropic drugs during breastfeeding, although the evidence has shown their relatively significant safety for the neonate [15–18].

Preventing this disorder is of great importance due to its unpleasant consequences for the physical and psychological health of the parents and particularly the neonate [19]. Furthermore, about 30 to 50 billion dollars are spent annually for the treatment of depression, and it also causes more indirect costs considering the inability to work [20].

One of the drugs that have been considered for the treatment of depression is ketamine [21]. Ketamine is an N-methyl-D-aspartate (NMDA) receptor antagonist and is considered an inexpensive, accessible anesthetic medicine [22]. Prescribing a dosage of ketamine that is lower than its anesthetic dose before anesthesia induction during caesarian sections would prevent severe hemodynamic changes along with appropriate analgesia induction; also, it does not have a decreasing effect on the respiration rate of the neonate’s [23].

Ketamine has a rapid anti-depressant effect in many patients resistant to treatment, although its level and stability of response is not predictable [24, 25]. Some studies have reported decreased symptoms of depression two hours after intravenous administration of low-dose ketamine with a two-week length of effect [26]. Although the effect of ketamine on major depression has been investigated and discussed in many studies, its effect on postpartum depression during general anesthesia for the caesarian section has rarely been investigated. Therefore, the present study was conducted to determine the effect of ketamine on preventing postpartum depression in women undergoing caesarian sections.

## Material and Methods

The study population for the present double-blind clinical trial included all the pregnant women who were a candidate for caesarian section referring to the educational centers of the Rafsanjan University of Medical Sciences. The sample size was calculated using the following formula and previous studies [27–29]. A primary error of 5%, a secondary error of 10%, a standard deviation of 4.1, and a score difference of 2.5 were considered for the 35 participants of each group.


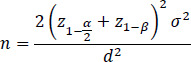


The inclusion criteria were having a low-risk pregnancy, being 18 to 35 years old, being a candidate for a caesarian section, being ASA (American Society of Anesthesiologists) class 1 or 2, (not having any underlying diseases such as ischemic heart diseases, diabetes mellitus or hypertension), not having any contraindication for receiving ketamine, and not having a history of drug abuse. Post-delivery hemorrhage, which required a blood transfusion, and the patient's unwillingness to continue the study were the exclusion criteria.

After receiving the approval of the University's Ethics Committee, data were gathered using a demographic characteristics questionnaire that investigated age, education, number of pregnancies, history of miscarriage, having a wanted or unwanted pregnancy, history of depression and satisfaction with life, and also the Edinburgh Postnatal Depression Scale (EPDS). This questionnaire consists of ten 4-choice questions so that its minimum score is 0 and its maximum score is 30. Mothers who would gain a score of 13 or higher are probably suffering from depression with various intensities, and they need further investigation to diagnose depression. Holden *et al.* reported that this questionnaire has a desirable validity and, using Cronbach's α, reported that its reliability is higher than 0.80 [30]. Ahmadi *et al.* reported a reliability of 0.70 among the Iranian population [31].

After obtaining written informed consent, eligible participants were divided into two groups (intervention and control) using simple randomization. The demographic characteristics form and Edinburgh scale were completed at the first stage and before the caesarian through an in-person interview. In the intervention group, along with Nesdonal (1–2 mg/kg of body weight), 0.5 mg/kg of body weight of ketamine was intravenously injected during the induction of anesthesia; the control group only received 3–5 mg/kg of body weight of Nesdonal intravenously during the induction of anesthesia. At the second and third stages of the study, which were two and four weeks after the caesarian section, the Edinburgh scale was completed again through phone calls. The gathered data were entered into the SPSS software (version 18) and analyzed using variance analysis with repeated measures and the Chi-square test.

## Results

**Figure 1.**
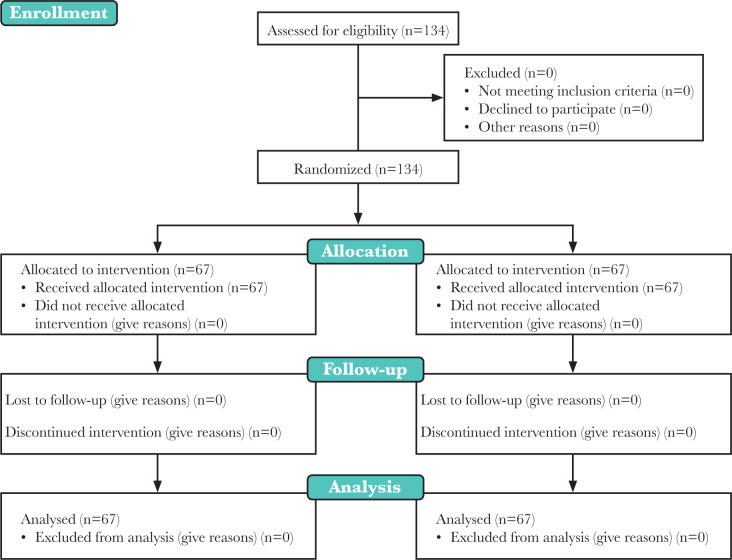
Consort flow diagram.

In total, 67 participants were studied in both groups (Figure 1). The mean age of the mothers in the Nesdonal group and the Ketamine-Nesdonal group was 27.4±4.09 and 28.24±4.81, respectively, and the difference between both groups was not statistically significant. Based on the results, both of the studied groups were similar regarding the number of pregnancies, having wanted or unwanted pregnancy, education, history of depression, and satisfaction with life (Table 1). The mean Apgar score in the first minute after delivery in the Nesodnal group was 7.30±0.63, and 7.82±0.68 in the Ketamine-Nesdonal group; in this regard, the difference between both groups was statistically significant (p > 0.001). Therefore, the Apgar score in the first minute was considered as a confounding factor in the model, and it was revealed that, despite this fact, there was a significant difference between both groups at different times regarding the score of depression (the interactive effect between time and Apgar score: P = 0.71, F = 0.35; the interactive effect between time and group: P < 0.001, F = 14.17). The Apgar score of all the neonates in the fifth minute was 10.

**Table 1:** The mean and standard deviation of the demographic characteristics of the participants in the intervention and control groups.

**Demographic characteristics**		**Intervention group**		**Control group**		**Independent t-test**	**P-value**
		**Mean**	**Standard deviation**	**Mean**	**Standard deviation**		
**Mother’s age**		27.40	4.09	28.24	4.81	-1.08	0.28
**Number of pregnancies**		2.06	0.85	2.21	0.98	-0.96	0.34
		**Frequency**	**Percent**	**Frequency**	**Percent**	**Chi-square test**	**P-value**
**Pregnancy**	**Wanted**	58	86.6	54	81.8	0.56	0.45
	**Unwanted**	9	13.4	12	18.2		
**Education**	**Under diploma**	5	7.5	4	6	2.23	0.33
	**Diploma**	17	25.3	25	37.3		
	**Bachelor’s degree or higher**	45	67.2	38	56.7		
**History of depression**	**Yes**	15	22.4	10	14.9	1.23	0.27
	**No**	52	77.6	57	85.1		
**Satisfaction with life**	**Excellent**	11	16.4	8	11.9	0.63	0.89
	**Good**	36	53.7	37	55.2		
	**Moderate**	16	23.9	17	25.4		
	**Poor**	4	6	5	7.5		

Results of variance analysis with repeated measures showed that in the Ketamine-Nedonal group, the mean score of depression was significantly lower four weeks after the caesarian section compared to two weeks afterward and also was significantly lower two weeks after caesarian section than before caesarian section (p > 0.001). In the Nesdonal group, the mean score of depression was increased two weeks after the caesarian section compared to before the caesarian section, but the difference was not statistically significant; in this group, the mean score of depression was significantly decreased four weeks after the caesarian section in comparison to two weeks after the caesarian section (p > 0.001). The mean score of depression had a significant difference between both groups, two and four weeks after the caesarian section (Table 2).

**Table 2:** Comparison of mean depression scores at different times between the two groups.

**Group** **Variable**	**Nesdonal group**		**Ketamine-Nesdonal group**		**Independent t-test and P-value**	**Variance analysis with repeated measures**	**P-value**
	**Mean**	**Standard deviation**	**Mean**	**Standard deviation**			
**Score of depression before caesarian section**	13.79	4.78	13.78	3.87	t = 0.02 p = 0.98	6.56	0.012
**Score of depression two weeks after caesarian section**	14.34	4.29	11.82	3.41	t = 3.77 p < 0.001		
**Score of depression four weeks after caesarian section**	13.09	3.79	10.84	3.48	t = 3.58 p < 0.001		
**Greenhouse-Geisser test**	F = 4.68		F = 52.52		-		
**P-value**	0.019		< 0.001		-		

## Discussion

The present study showed a significant difference between the intervention and the control groups at different times regarding the score of depression except for before the caesarian section, meaning that the scores of depression two and four weeks after the caesarian section were significantly lower than the control group. The depression scores in the intervention group at the three studied times were significantly lower and descending; the scores were lower than the cut-off point of the Edinburgh scale for the possible depression diagnosis two and four weeks after the caesarian section. In the Nesdonal group, the score of depression was increased two weeks after the caesarian section compared to before the intervention, but the difference was not statistically significant. However, the score of depression was significantly decreased in the control group four weeks after the caesarian section compared to two weeks after the caesarian section. Nevertheless, this score was still higher than 13 and considered possible depressed according to the Edinburgh scale and required more investigation.

Although the difference in the Apgar score of the neonates in the first minute was statistically significant between the two groups, the difference was not clinically significant, and both groups received a score of 7 and had similar categorization regarding their need for resuscitation. However, considering it as a confounding factor in the investigation model, it had no effect on the score of depression. In a systematic review by Hessen *et al.* in 2015, ketamine did not have an effect on the Apgar score either [32].

In a study that was titled “the effect of Ketamine on postpartum depression in women undergoing caesarian section”, Jianxin *et al.* also found similar results in 2015 and reported the positive effect of ketamine on preventing postpartum depression [33].

Unlike the results of the present study, the results of the study conducted by Xu *et al.* in 2017 showed that ketamine had no effect on preventing postpartum depression [34]. The reason for not finding any effect was probably the lower dose of ketamine (0.25 mg/kg of body weight) in comparison to the present study (0.5 mg/kg of body weight) because Jianxin *et al.* also reported similar results and a positive effect in their study with a higher dose of ketamine (4 mg/kg of body weight) than the dose used in the present study.

In 2016, Xia *et al.* evaluated the effect of chronic stress before pregnancy on postpartum depression and compared the effect of fluoxetine and ketamine in rats. They reported that acute ketamine had improved the molecular signaling disorder, and behavioral flaws in rats with postpartum depression were corrected rapidly and stably compared to the poor treatment with chronic fluoxetine [35]. The greater effectiveness of ketamine compared to fluoxetine in the treatment of postpartum depression was a notable point in this study because selective serotonin reuptake inhibitors (SSRIs), during pregnancy and after delivery are potentially capable of affecting the development process of the neonate due to passing the placenta and being active in the breast milk. However, the weight of the effect of the mother’s depression or these drugs on the development of the neonate has not been determined [36]. Therefore, in the case of the positive effect of ketamine on postpartum depression, there are no concerns.

Regarding the effect of ketamine on postpartum depression, unlike its effect on major depression, the studies are limited. However, considering the extensive confirmation of the effect of ketamine for the treatment of major depression and suicidal thoughts in various studies [37–41], it would not be too much to expect a positive effect from it on postpartum depression.

Various studies have been conducted in the midwifery and gynecology setting on issues other than postpartum depression, such as its analgesic effect after caesarian sections and also analgesia during labor, and most of the studies have confirmed the positive effect of ketamine.

Appropriate pain relief after a caesarian section is essential for mother-neonate bonding, early recovery of the mother, and early discharge from the hospital. Most of the conducted studies in this regard studied ketamine’s spinal administration, which had appropriate effectiveness and decreased the need for pain killers after the caesarian section [32].

Inducing appropriate analgesia during labor with a safe, affordable, simple method with the least maternal and fetal complications that would not affect the process of labor is considered an ideal method, and some studies have found ketamine appropriate for this purpose [42, 43].

Considering the reported advantageous effects of ketamine in the conducted studies such as analgesia [44, 45], analgesia during labor [42, 46] after caesarian section and vaginal delivery [47], safety during labor and caesarian section[48], and its relatively known effect for the treatment of major depression, if its effect on preventing postpartum depression would be confirmed through another clinical trial, it would become a multipurpose appropriate option in gynecology and midwifery departments. This effect could especially be considered in people who are more prone to postpartum depression.

## Conclusion

Postpartum depression is a disabling common psychosocial disorder and could have destructive effects on the mother, neonate, family, and society. The present study evaluated the effect of a single dose of intravenous ketamine during a caesarian section on preventing postpartum depression, considering the conducted studies on the effect of ketamine on treating major, treatment-resistant depression. Based on the results of the present study, it could be concluded that a single dose of ketamine in women who are a candidate for a caesarian section could be effective in preventing postpartum depression. Since ketamine is inexpensive and available, it could mainly be used for this purpose in women who are more prone to postpartum depression. However, extensive clinical trials should be conducted in this regard.

## Acknowledgments

### Ethical approval

The approval for this study was obtained from the Ethics Committee of the Rafsanjan University of Medical Sciences (approval ID: IR.RUMS.REC.1395.27). The trial was registered at the Iran Clinical Trial Registration Center (reference number: IRCT2016122726971N2).

### Consent to participate

Written consent was obtained from each participant.

### Conflict of interest

The authors declare that there is no conflict of interest.
